# Relationships between individual attitudes and occupational stress. A cross-sectional study

**DOI:** 10.3934/publichealth.2025030

**Published:** 2025-05-26

**Authors:** Nicola Magnavita, Francesco Marcatto, Igor Meraglia, Giacomo Viti

**Affiliations:** 1 Department of Life Sciences and Public Health, Università Cattolica del Sacro Cuore, 00168 Roma, Italy; 2 Department of Life Sciences, University of Trieste, 34128 Trieste, Italy

**Keywords:** work annoyance, work engagement, overcommitment, social capital, psychosocial stress, health surveillance, health promotion, stress prevention, workplace

## Abstract

Understanding the impact of work attitudes on occupational stress is essential to promote employee wellbeing and productivity. This study investigates the associations between different work attitudes (work annoyance, individual social capital, overcommitment, and work engagement) and the perceived stress. A cross-sectional survey conducted among 1290 employees from various occupational sectors assessed their attitudes and stress levels using validated psychometric scales. Statistical analyses, including a hierarchical regression and a moderation analysis, examined the predictive value of each attitude and the potential buffering role of social capital. The results indicate that work annoyance and overcommitment are positively associated with stress, which suggests that perceiving job conditions as frustrating and investing excessive effort without the appropriate rewards contribute to psychological strain. Conversely, social capital and work engagement exhibit a protective effect, with workplace relationships and a positive approach to work mitigating stress levels. Moreover, social capital moderates the relationship between overcommitment and stress, thus highlighting its buffering effect. These findings emphasize the importance of fostering a supportive work environment that reduces negative attitudes while promoting engagement and social cohesion. Organizational interventions aimed at improving workplace relationships, recognizing employees' contributions, and encouraging a balanced work culture could be effective strategies to enhance the workers' wellbeing and mitigate occupational stress.

## 1. Introduction

Work attitudes indicate assessments of a job that encompass the emotions, convictions, and commitment of an individual employed in that position [1]. Owing to their predictive efficacy concerning job performance, job happiness, and workplace mental health, these research areas are among the oldest, most widely recognized, and impactful in organizational psychology [1],[2]. In fact, research demonstrates that subjective judgments of job characteristics act as situational precursors to job attitudes, and several models have been proposed to elucidate their impact on psychological outcomes in the workplace. Hackman and Oldham's Job Characteristics Theory (JCT) outlines five basic subjective job elements: skill variety, task identity, task significance, autonomy, and feedback [3]. These aspects anticipate important psychological states, which, in turn, predict personal and professional success.

Occupational stress is the pressure that work exerts on an individual. Numerous stressors can be present in any work environment. However, the role of these factors largely depends on the individual. The same stimulus may have different effects on people: it may be negative for one, neutral for another, and positive for a third. This individual variability explains why it is often preferable in occupational medicine to measure the perception that workers have of their work, rather than evaluate the extent and duration of stress factors in absolute terms [4]. The negative effects of stress are generally referred to as “distress”. This is a condition that is not yet a disease but, if exposure to occupational stressors is too intense or prolonged, it can result in impaired physical and mental health and behavioral disorders. Work demands, poor work environment, and a poor work life balance are among the main causes of distress and burnout, which is a syndrome characterized by three factors: emotional exhaustion, depersonalization, and a perceived lack of personal fulfilment [5]–[7].

Occupational stress is only one of the psychosocial stressors to which everyone is exposed to because of life events. To correctly measure the level of occupational stress, among the various available models, it is important to choose the one that best expresses the particular characteristics of the work investigated. For example, during the first industrial revolution, when many tasks required intense physical effort, there was a pace dictated by machines; the most suitable model was Karasek's job strain (1979), in which stress was conceived as a weighted relationship between the workload (demand) and the ability to control the process (skills plus discretion). To this model, Swedish researchers added the evaluation of social support, which is an important moderating factor of stress [8],[9]. With the development of services and the second industrial revolution, many jobs were more effectively described using Siegrist's effort-reward model [10], which evaluates the discrepancy between the effort each worker makes in order to work and the reward they receive in exchange. A complementary model is the job demands-resources (JD-R) theory [11],[12], which considers the buffering role of various job resources on the impact of job demands on distress and burnout. The quality of the results obtained depends on the researcher's choice of the most appropriate model for each research study.

The literature on the effects of occupational stress includes a multitude of contributions, which were often collected in systematic reviews and meta-analyses. Exposure to occupational stress is associated with an increased risk of hypertension [13],[14], dyslipidaemia [15], coronary heart disease [16]–[19], stroke [20], diabetes [21]–[24], and metabolic syndrome [25]–[29]. Work related stress exposure is also associated with dementia and cognitive impairment [30], with an increased risk of lung, colon, and esophageal cancer [31]. Moreover, there is some evidence that job stressors may be related to suicidal outcomes [32]. Furthermore, work related stress is associated with mental disorders [33], insomnia and sleep impairment [34]–[36], depression [37],[38], and an increased risk of psychotropic medication use [39]. Stress measured by questionnaires has a significant association with objective indicators such as heart rate variability [40], cortisol buildup in hair [41], immune parameters [42], and those related to the hypothalamic-pituitary-adrenal axis [43].

This brief and partial review of the effects of work-related stress explains why it is important to identify the causes and know how to prevent them. The way each worker relates to his or her work tasks is important in terms of the perception of work-related stress. According to the JD-R model, environmental and organizational stressors are the demands employees must face, while their attitudes reflect the available resources. This general principle requires identifying attitudes that could be linked to stress. The term “job attitudes” does not have a universally accepted definition, hence different scholars have given it varied interpretations. Historically, research has mainly focused on defining and quantifying work involvement [44] and studying the connection between work organization and employee attitudes [45]. Work engagement and overcommitment are two commonly used variables to assess people's emotional attachment to or involvement in their work; the former is associated with positive outcomes, while the latter has detrimental consequences for health and well-being. Overcommitment and work engagement are two different constructs, even though they are moderately associated [46]. Despite drawing from the same pool of psychological resources, these two measures have rarely been investigated at the same time. Moreover, the relationship between the employees' attitudes and work-related stress necessitates a consideration of workforce involvement and interactions, which is often assessed through variables such as social capital. Social capital denotes the resources accessible to an individual via their interpersonal workplace connections. It is a complex and dynamic psychological construct that highlights the importance of interpersonal relationships, the opportunities accessible through interactions, and social network links. It is influenced by the organization of social relationships (structural factors), the level of interpersonal connections, which encompass emotional bonds, reliance, and respect (relational factors), and the shared interpretation and acceptance through social groups (cognitive factors) [47],[48]. A recent area of study focused on a novel attitude toward work: preliminary intolerance toward common work demands, termed “Work Annoyance”. Work annoyance refers to the degree of irritation or frustration that employees experience in response to recurrent conditions of work-related discomfort such as the need to work beyond regular hours, or the high cognitive demands of the job, which include mental workload, task complexity, and the necessity for ongoing learning [49]. Research indicates that work engagement and work annoyance possess distinct determinants and effects [50].

Overcommitment is a concept proposed by Siegrist in the Effort–Reward Imbalance model to account for a personal inclination that explains why certain individuals invest excessive effort in their work despite the lack of appropriate rewards or incentives. Overcommitment refers to employees who exhibit a motivational pattern that compels them to labor excessively, thus rendering themselves incapable of disengaging from their responsibilities. These employees have a strong desire for validation and a distorted perception of their job requirements and accessible coping mechanisms. According to Siegrist [10], overcommitted employees display specific attitudes, behaviors, and emotions that lead them to desire and make excessive attempts to be respected and appreciated. These endeavors induce them to take on an exaggerated number of challenging activities and exert excessive efforts to complete them [51]. Additionally, they may have an incorrect perception of resources and demands [52]; this may explain why they endure straining jobs [53]. Because their efforts are disproportionately high, overcommitted workers are more likely to be affected by subjectively poor rewards, which may lead to frustration [51]. Important correlations have been demonstrated between overcommitment and burnout and a reduced well-being [54],[55]. A longitudinal study showed that overcommitment is a preliminary factor in the onset of alcohol abuse [56] and is associated with severe psychological distress in students [57]. In the effort/reward imbalance model, overcommitment constitutes the intrinsic component of stress; for this reason, it is linked to the effects of stress on health [58].

While excessive commitment to work is a negative factor for the well-being of workers, “work engagement” or the constructive and satisfying state of mind that promotes the well-being is considered a positive attitude. Both overcommitment and engagement can be seen as forms of heavy work involvement [59]. However, they have a different relational attitude. Work engagement expresses the degree to which a person is willing to promote connections between self and job [60]. It is composed of three related cognitive, emotional, and physical personal resources [61]. On the contrary, the overcommitted worker has a personal goal: that of promoting their own position through a maximum commitment to work.

Work attitudes can be considered the result of two factors: the characteristics of each individual employee and environmental pressures. In fact, an individual's disposition towards work can be influenced by the organizational climate; this is the basis for stress control programs. Social capital, which integrates social support, social networking, and social cohesion [62], is an example of this multiple origin. It stems from each employee's sociable traits, the leadership style, and the atmosphere of the workplace. Individual social capital and group social capital are the two broad conceptualizations which contribute to forming an attitude known as social capital [63]. Several studies have contributed to demonstrating that social capital is associated with a workers' well-being and productivity [64]–[67] and a reduced risk of job strain [68],[69]. Additionally, it has been shown that this variable mediates between other work attitudes and work abilities [70].

The important role that psychosocial stress plays in the physical and mental health of workers led us to investigate the relationship between attitudes and work-related stress. Therefore, we assessed the workers' attitudes and occupational stress in all those who underwent health surveillance in the workplace in companies assisted by our university to study the relationship between these attitudes and work-related stress.

Based on the literature data, the hypotheses we formulated were as follows:

1. Work annoyance is positively associated with perceived stress;

2. Social capital is negatively associated with perceived stress;

3. Overcommitment is positively associated with perceived stress;

4. Work engagement is negatively associated with perceived stress; and

5. Social capital can moderate the relationships between negative work attitudes and stress.

## 2. Materials and methods

### 2.1. Sample

In compliance with legal requirements, a medical examination was conducted for employees exposed to occupational risks, during which 1290 workers completed a questionnaire that included personal data and assessments of individual working attitudes and work-related stress. The workers were engaged in various sectors, including industry, commerce, health, social assistance, and administration. The eligibility criteria included being summoned for the periodic workplace medical examination and consenting to participate in the survey by signing the informed consent form. Workers who opted to not participate or who failed to complete the survey were excluded from the study.

### 2.2. Measures

#### 2.2.1 Work annoyance

Work Annoyance (WA) refers to the degree of irritation experienced by employees in relation to various occupational elements. It is quantified by means of the Work Annoyance Scale [42], which is a self-reported instrument comprised of nine items. The participants assessed their annoyance levels related to specific work characteristics on an 11-point Likert-type intensity scale, where 0 indicates ‘No annoyance’ and 10 signifies ‘Utmost annoyance.’ The items refer to commonplace working conditions, such as night shifts and cognitive demands, alongside the requirement to learn a new language, which workers often identify as sources of dissatisfaction. The nine questions yielded a final score that ranged from 0 to 90. In this study, Cronbach's alpha coefficient was 0.797.

#### 2.2.2 Workplace social capital

The measurement of Workplace social capital was conducted using the 8-item scale developed by Kouvonen et al. [71], which evaluates items on a 5-point scale, where 1 represents “fully disagree” and 5 signifies “fully agree.” The final score ranged from 8 to 40. The reliability, as measured by Cronbach's alpha, was 0.929.

#### 2.2.3 Overcommitment

Overcommitment, which is an intrinsic component of the effort–reward model of stress [72], was assessed using the Italian version [73] of Siegrist's short form questionnaire [74]. Six items were evaluated using a 4-point scale, which yielded potential scores between 6 and 24. The value of Cronbach's alpha was 0.748.

#### 2.2.4 Work engagement

Work engagement was assessed using the Italian adaptation [75] of the Utrecht work engagement scale-UWES [76]. The abbreviated questionnaire comprised of 9 items, with scores ranging from 0 to 6. The questionnaire included three components: Vigor, Dedication, and Absorption, each associated with three items. The Cronbach's alpha values for the components were 0.842 for Vigor, 0.899 for Dedication, and 0.719 for Absorption. The total score, which encompassed all 9 items, yielded a Cronbach's alpha of 0.907.

#### 2.2.5 Work-related stress

Work-related stress was measured using an abbreviated Italian version [77] of the Effort/Reward Imbalance Questionnaire based on Siegrist's model [74]. Three items for the effort variable and seven for the reward variable were included in the condensed version of the survey. The resulting sub-scales ranged between 3 and 12 (effort) and 7 and 28 (reward), respectively, because all items included responses that were evaluated on a 4-point Likert scale. If the effort/reward imbalance (ERI), which is a weighted relationship between the two variables, was more than one, then it indicated a state of discomfort. In this study, Cronbach's alpha for the effort sub-scale was 0.780, while Cronbach's alpha for the reward sub-scale was 0.706.

### 2.3. Ethics approval of research

The study was conducted in accordance with the Helsinki Declaration. The participants provided informed consent by signing their personal health document. In accordance with the International Commission on Occupational Health's (ICOH) code of ethics and principles of confidentiality in occupational medicine, the participants consented to the analysis of their personal data on condition that the results were shared in an anonymous form. Ethic approval was granted by the Catholic University Ethics Committee (ID 3008, 5 June 2020). Due to the cross-sectional nature of the study, the findings were solely based on completed survey responses.

### 2.4. Statistical analyses

The scores resulting from the surveys were initially analyzed using mean, median, and standard deviation. The Kolmogorov-Smirnov and Shapiro-Wilk tests were used to assess the normality of the distribution. In cases of considerable deviation from normality, we reported the median value and used principally non-parametric testing, even though the sample size permitted the use of conventional parametric tests, as per Lumley et al. [78].

Stepwise multiple hierarchical linear regression models were used to evaluate the predictive significance of each distinct job-related variable on work stress. In Model 1, we designated Work Annoyance as an independent variable and controlled age and sex. We subsequently incorporated social capital as a predictor. In the third model, we incorporated Overcommitment. In the final model, we added the elements of work engagement.

To assess the association between overcommitment, social capital at work, and work stress, we initially examined the correlation between these continuous variables by utilizing the Pearson correlation coefficient. After confirming a substantial correlation between the three variables, we assessed their relationship by proposing that overcommitment could serve as a predictor, social capital might function as a moderator, and work stress was the dependent variable. We created two moderation analysis models: the first included sex and age as covariates; and the second included work annoyance and work engagement as cofactors.

We used IBM/SPSS Statistics for Windows, Version 28.0 (IBM Corp.: Armonk, NY, USA), and PROCESS v.4.1 by Andrew F Hayes (2023) for the analysis.

Data are deposited on Zenodo doi: https://doi.org/10.5281/zenodo.15041736.

## 3 Results

Out of a total population of 1522 individuals (participation rate 84.8%), which was composed of all workers described as “at occupational risk” in their companies, 1290 employees (485 males, 37.6%; 805 females, 62.4%) participated in the study. The sample characteristics are listed in Table 1. Given the slight difference between the means and medians, the significance of the Shapiro-Wilks and Kolmogorov-Smirnov tests was probably due to the large sample size.

**Table 1.** Age, attitudes and work-related stress of the observed sample.

**Variable**	**Range**	***Mean* ± *S.D*.**	**95% *CI***	**Median**
Age	20–71	45.86 ± 10.76^1^	45.27; 46.45	46
Work annoyance	0–90	32.44 ± 17.83^1^	31.45; 33.42	33
Social capital	8–40	26.30 ± 8.50^1^	25.84; 26.77	26
Overcommitment	6–24	13.26 ± 3.52^1^	13.07; 13.45	13
Work engagement	0–54	37.15 ± 9.42^1^	36.64; 37.67	37
Effort	3–12	7.46 ± 2.28^1^	7.34; 7.59	7
Reward	7–24	18.95 ± 3.73^1^	18.75; 19.15	19
ERI	0.29–4.0	0.98 ± 0.45^1^	0.96; 1.01	0.93

Note: ^1^ Kolmogorov-Smirnov and Shapiro-Wilk tests, *p* < 0.001. *S.D*. standard deviation. 95% *CI* confidence interval 95%.

The results indicated a substantial link between all work attitudes. Compared to their male colleagues, females manifested a greater annoyance, as well as a diminished social capital and a slightly higher Overcommitment. Age was significantly correlated with Work Annoyance and Overcommitment, and inversely correlated with social capital, work engagement, and stress. Increased levels of Annoyance correlated with elevated levels of Overcommitment and diminished levels of social capital, work engagement, and Stress. Social capital was inversely correlated with Overcommitment and Stress and positively related to work engagement. Overcommitment was strongly correlated with Stress and inversely correlated with work engagement. Work engagement was inversely related to Stress (Table 2).

**Table 2.** Bivariate correlation between the variables used. Spearman test (upper triangle) and Pearson test (lower triangle).

**Project**	**Female**	**Age**	**Annoy.**	**SocCap**	**Overcom.**	**Engag.**	**ERI**
**Female**	1.000	0.026	0.130**	–0.083**	0.059*	–0.002	–0.001
**Age**	0.023	1.000	0.198**	–0.202**	0.143**	–0.092**	–0.198**
**Annoy.**	0.135**	0.210**	1.000	–0.218**	0.183**	–0.415**	–0.253**
**SocCap**	–0.081**	–0.210**	–0.231**	1.000	–0.286**	0.371**	–0.397**
**Overcom.**	0.055	0.145**	0.196**	–0.289**	1.000	–0.179**	0.533**
**Engag.**	–0.001	–0.101**	–0.417**	0.387**	–0.186**	1.000	–0.344**
**ERI**	–0.009	0.181**	–0.245**	–0.386**	0.548**	–0.331**	1.000

Note: * Correlation is significant at the 0.05 level (2-tailed). ** Correlation is significant at the 0.01 level (two-tailed)

Hierarchical linear regression models indicated a positive correlation between Work Annoyance and Work Stress (Model I, *p* < 0.001). Social capital correlated with diminished Work Stress; in Model II, which incorporated Work Annoyance and social capital as predictors, both exhibited significant associations with stress (*p* < 0.001). Overcommitment was strongly correlated with elevated stress (*p* < 0.001); incorporating this variable into Model III allowed Work Annoyance and social capital to maintain their status as major predictors of stress. Work engagement exerted a significant beneficial effect on stress in Model IV (Table 3). The final model of the multiple regression equation indicated that Overcommitment is the main factor of occupational stress, while social capital and work engagement tend to reduce the workers' perceived stress. The model that included all attitudes accounted for about 40% of the total variance of work-related stress.

**Table 3.** Effect of individual attitudes on work-related stress (ERI). Hierarchical linear regression models.

**Variable**	**Model I**		**Model II**		**Model III**		**Model IV**	
	**Beta**	***p***	**Beta**	***p***	**Beta**	***p***	**Beta**	***p***
Sex (Female)	–0.043	0.114	–0.059	0.023	–0.070	0.002	–0.059	0.009
Age	–0.136	<0.001	0.076	0.004	0.043	0.066	0.052	0.024
Work annoyance	–0.222	<0.001	0.156	<0.001	0.095	<0.001	0.035	0.169
Social capital			–0.350	<0.001	–0.237	<0.001	–0.182	<0.001
Overcommitment					0.462	<0.001	0.457	<0.001
Work engagement							–0.164	<0.001
Adjusted *R* square	0.077		0.189		0.378		0.396

A moderation analysis showed that social capital moderates the effect of Overcommitment on Work Stress. The interaction between the two variables was significant, both in the model adjusted for age and sex (Model I, *p* < 0.05) and in the one including all the work attitudes (Model II, *p* < 0.01) (Table 4).

**Table 4.** Moderating effect of social capital in the interaction between overcommitment and work stress.

**Variables**	**Model I^1^**		**Model II^2^**	
	**Beta**	***p***	**Beta**	***p***
Overcommitment	0.079	<0.001	0.080	<0.001
Social capital	–0.003	0.446	0.002	0.707
Overcommitment × Social capital	–0.001	0.021	–0.001	0.006

Note: ^1^ Adjusted for age and sex, ^2^ Additionally adjusted for work annoyance and work engagement.

## 4 Discussion

This study demonstrated that both intolerance for common problems encountered in work environments and excessive work commitment are associated with perceived stress. In contrast, attention to social relationships and the appreciation of one's work are associated with a reduction in the perceived stress. By comparing the different attitudes towards one's work, it is possible to observe that the attitudes in which the worker focuses on themself in order to defend themself from possible occupational adversities (work annoyance) or make the most of their own abilities (overcommitment) have a negative effect, while the attitudes directed towards others, such as social capital and work engagement, have a positive effect on mental health.

All the hypotheses that we made and that led us to design this survey were confirmed. Work annoyance was found to be positively associated with perceived stress, while social capital was negatively associated with stress. Overcommitment was a significant determinant of perceived work-related stress, while positive motivation (work engagement) was negatively associated with perceived stress. These results correspond to the first four hypotheses that we formulated. The fifth hypothesis, namely that social capital can moderate the relationships between negative work attitudes and stress, was also confirmed, and this opens perspectives for interventions to promote mental health in the workplace.

Work annoyance was found to have a significant association with stress, which suggests that employees who perceive their usual working conditions as frustrating may experience increased occupational strain. This aligns with previous findings which indicated that negative perceptions of job characteristics can amplify stress responses [79],[80]. Similarly, overcommitment emerged as a key predictor of stress, thus reinforcing the idea that excessive work involvement may lead to psychological strain and a reduced well-being [54],[55].

On the other hand, the results showed that social capital and work engagement act as protective factors against stress. Social capital, which reflects the quality of workplace relationships and support systems, and therefore depends both on the personal attitude for social relations and on the relational climate of the work group, showed a negative correlation with stress; this indicates that stronger social bonds can help employees cope with workplace challenges. Furthermore, it moderates the effect of work annoyance and overcommitment. Work engagement, which is an attitude characterized by vigor (having a lot of energy and mental toughness while working, being willing to put effort into one's work, and persevering through challenges), dedication (feeling a sense of importance, excitement, inspiration, pride, and challenge), and absorption (being totally focused and absorbed in one's work, which causes time to fly by and makes it difficult to separate oneself from work) were also negatively associated with stress, which highlights the role of positive psychological states in shielding against job-related strain. These findings agree with the literature in studies that have generally considered the effect of single variables. The inverse relationship between work engagement and occupational stress emerged in all systematic reviews on these topics [81]–[85]. Work engagement is considered a factor capable of mediating the relationship between environmental tensions (stress) and the resulting state of exhaustion (burnout), which is on a par with resilience and coping strategies [86]. Organizational policies are significantly associated with employee engagement and consequently influence the level of employee strain [87]. In fact, workplace health promotion programs based on mindfulness have been shown to increase work engagement and reduce the levels of strain [88]. Positive attitudes permit better management of chronic pathologies; for example, high levels of work engagement are associated with appropriate physical activity, trigger avoidance, and good self-management in asthmatic workers [89]. Work engagement increases with chronological age and moderates the relationships between various job-related psychological and work-environmental factors; consequently, it is associated with working beyond retirement age [90]. Conversely, overcommitment is associated with the poor management of chronic conditions. More than psychosocial stress, overcommitment is a major determinant of musculoskeletal disorders [91] and is linked to an increased allostatic load in healthcare workers [92]. A longitudinal study of Swedish public sector workers showed that overcommitment combined with job stress predicted a poor work ability [93]. Occupational stress is significantly associated with a reduced quality of life, and overcommitment is a mediator of this relationship [94]. A recent study showed that overcommitment was associated with increased cortisol levels solely in workers who were in great need of rest; this is because excessive commitment forces one to work against fatigue in conditions of strain [95].

Our study considered several attitudes simultaneously, which allowed us to observe their relationships. Work annoyance can be considered as a preliminary state to distress, which can be easily modified and overcome by a high social capital. Work engagement and overcommitment have much greater weight in determining the perceived stress; however, even in this case, a high social capital can moderate the effects. The relationship between the different attitudes is rather complex. According to Bereznowski et al. [96], work engagement could lead to compulsive work (work addiction or overcommitment), and this could determine a pathological state of psychological exhaustion or burnout. Longitudinal analyses conducted using data from the Third German Sociomedical Panel of Employees on German employees aged 40–54 years indicated that overcommitment is significantly associated with mental health and probably acts as a mediator between stress and poor mental health [97].

Social capital has been widely studied as a protective factor in the workplace, since it contributes to employee well-being and shields against stress. Research suggests that high levels of social capital are associated with lower psychological distress and an improved job satisfaction, since strong interpersonal relationships and mutual trust create a supportive work environment that encourages collaboration and reduces feelings of isolation [98],[99]. Social capital is also positively linked to work engagement, as employees who perceive a sense of belonging and shared purpose within their organization are more likely to demonstrate commitment and enthusiasm toward their work [100]. Conversely, lower social capital has been associated with heightened stress levels and an increased risk of burnout, particularly in high-demand occupations where interpersonal support is crucial [101]. Low workplace social capital is also a strong predictor of depression, as demonstrated in a study of Finnish public sector employees [102], is associated with binge drinking in older workers, as highlighted in a Health and Retirement US study [103], and with polypharmacy, as shown in a Survey of Health, Ageing and Retirement in Europe (SHARE) [104]. Conversely, high social capital is associated with successful ageing according to the China Health and Retirement Longitudinal Study (CHARLS) [105]. Based on these studies, it would be expected that workers with longer employment duration experience greater social capital; future research could test this hypothesis. Furthermore, studies have revealed the protective effect of workplace social capital in reducing work-home conflict [106],[107], since social capital enhances an employees' ability to balance professional and personal responsibilities by fostering mutual support, trust, and shared resources within the workplace. Obviously, organizations cannot act on the individual predisposition to develop relationships; however, they can foster an organizational climate in which the bonds between employees are strengthened. A supportive social environment leads to greater flexibility, improved communication, and reduced role conflicts, which ultimately promotes a better work-life balance and reduces stress related to competing demands.

By evaluating the role that different attitudes play in occupational stress, this study provides useful indications to plan interventions aimed at reducing work-related stress. Work organizations should aim to develop attitudes in workers that reduce the perceived stress and counteract the tendency to intrinsic stress or overcommitment. Management should devote special attention to develop programs that can increase social capital and the sense of belonging to the social group; an attachment to work should be rewarded, but employees should not be urged to abandon family and personal interests. Leadership plays a fundamental role in this process, as leaders shape the workplace culture, influence social interactions, and set the tone for how employees engage with their work. Transformational and inclusive leadership approaches that emphasize empowerment, collaboration, and shared purpose have been shown to strengthen social capital by fostering trust, reciprocity, and mutual support among employees [108],[109]. By promoting open communication, recognizing employees' contributions, and ensuring equitable participation in decision-making, managers can help create a work environment that reduces stress and enhances engagement [110]. Furthermore, leaders who encourage a healthy separation between professional and personal life contribute to reducing overcommitment and preventing stress-related exhaustion [111].

Only a small part of health promotion interventions (around 7%, according to the WHO, 2018) are developed in the workplace. Among these, an increasing number concern interventions with positive aims, such as measures to increase health and well-being rather than combating mental illnesses. This type of intervention offers greater prospects of success compared to traditional ones [112]–[114]. Moreover, health promotion programs that incorporate aspects of play or competition (gamification) achieve longer lasting results and are more sustainable [115]. A realistic review of the literature on workplace health promotion that included only studies based on validated instruments verified by Cochrane documented the importance of employee engagement in interventions on mental health improvement [116]. Work engagement mediates the relationship between stress and health perceived by the workers [117]. Furthermore, it is interesting to note that a positive attitude towards work can be associated with other salutogenic behaviors, such as a healthy diet [118] and physical activity [119].

Some organizational interventions aimed at reducing occupational stress have been applied in different workplaces. Studies have shown that interventions which focus on job redesign—such as increasing autonomy, clarifying roles, and reducing excessive workload—can significantly decrease stress and improve job satisfaction [9],[120]. Implementing flexible work arrangements, including remote work options and adjustable schedules, has also been linked to lower stress levels and an improved work-life balance [121]. Moreover, fostering a supportive work environment through peer-support programs, mentoring initiatives, and leadership training has been found to enhance social capital and reduce workplace stressors [122]. An analysis of the sixth European Working Conditions Survey which involved around 30,000 employees in 35 European countries showed that a better supervisor behavior quality was associated with increased workplace social capital [123]. Psychological interventions, such as coping skills and stress management training, have gained empirical support for their effectiveness in helping employees manage stress more effectively [124],[125]. Furthermore, the use of multimodal comprehensive interventions that combine individual and organizational-level actions, proved to be more effective than unidimensional programs [126] in reducing workplace stress and improving an employees' well-being.

Although this study was based on Siegrist's Effort-Reward Imbalance (ERI) model, the findings align well with the Job Demands-Resources (J-DR) model [127]. This model suggests that job demands and job resources are both vital for employee well-being, thereby exerting distinct and complementary effects. Job demands, such as work annoyance and overcommitment, contribute to health impairment by depleting an employees' energy and leading to strain and burnout. Work annoyance results from frustration due to occupational challenges, whereas overcommitment reflects an excessive investment in personal resources, thus making employees more vulnerable to stress-related impaired health. Conversely, occupational resources, such as social capital and work engagement, have a dual role: they provide a motivational boost that enhances employee well-being and performance, while simultaneously acting as a buffer against the negative impact of job demands. In more specific terms, by fostering supportive relationships in the workplace, social capital enhances an employees' ability to cope with stressors, while work engagement, characterized by vigor, dedication, and absorption, promotes job satisfaction and psychological well-being. Our moderation analysis further supports this interpretation, as social capital was found to buffer the negative effects of overcommitment on stress, which is consistent with the J-DR model's emphasis on the protective role of occupational resources.

Possible developments of the research could consider the evolution of attitudes in workers over time, which depends on numerous socio-economic and cultural factors, in a similar way to how the risk acceptability criterion, which is the basis of risk management, varies over time [128]. It is likely that social capital and overcommitment/engagement also significantly change over time due to important changes in the social structure, for example, through the work-devotion schema and its crisis [129],[130]. The strengths of this study include the investigation of the effects of different job attitudes on occupational stress - a topic still not fully studied in the literature - and the use of a large and heterogeneous sample that enables findings to be generalized across different occupational sectors. That said, some limitations must be acknowledged. First, the cross-sectional design precludes causal inferences. While associations between variables are robust, it is unclear whether work attitudes drive stress levels or vice versa; thus, longitudinal studies are needed to establish causality. Second, self-reported data may be subject to biases such as social desirability and recall bias. Lastly, while the study focused on attitudes as potential stress determinants, other factors not included here, such as the organizational climate, social support, and job security, might also play a role.

## 5 Conclusions

This study, which contributes to the understanding of how different work attitudes influence occupational stress, underlines the role of individual perceptions of stress experiences and well-being in the workplace. The findings suggest that attitudes which focus on self-protection from occupational adversities (work annoyance) and self-enhancement (overcommitment) may increase stress levels, while attitudes which foster social connections (social capital) and positive engagement with work may serve as protective factors.

Given these results, organizations should implement targeted interventions that address both individual attitudes and workplace conditions. Strategies to reduce work annoyance could include enhancing the job design to minimize unnecessary frustration, improving communication to clarify expectations, and providing employees with greater autonomy over their tasks. Preventing excessive work involvement requires the fostering of a healthy work culture that discourages overcommitment, promotes work-life balance, and ensures that employee efforts are appropriately recognized and rewarded. Moreover, strengthening social capital through social cohesion initiatives, mentorship programs, and leadership practices that encourage collaboration can help create a more supportive work environment. Finally, enhancing work engagement through meaningful job roles, opportunities for professional growth, and recognition of employees' contributions can further contribute to the overall well-being and productivity.

## Use of AI tools declaration

The authors declare they have not used Artificial Intelligence (AI) tools in the creation of this article.

## References

[1] Judge TA, Kammeyer-Mueller JD (2012). Job attitudes. Annu Rev Psychol.

[2] Shimmin S (1975). People and work: Some contemporary issues. Br J Ind Med.

[3] Hackman JR, Oldham GR (1976). Motivation through the design of work: Test of a theory. Organ Behav Hum Perform.

[4] Marcatto F, Di Blas L, Luis O (2022). The perceived occupational stress scale: A brief tool for measuring workers' perceptions of stress at work. Eur J Psychol Assess.

[5] Zhou AY, Panagioti M, Esmail A (2020). Factors associated with burnout and stress in trainee physicians: A systematic review and meta-analysis. JAMA Netw Open.

[6] Shoman Y, Rousson V, Bianchi R (2022). Holistic assessment of factors associated with exhaustion, the main symptom of burnout: A meta-analysis of longitudinal studies. Int J Environ Res Public Health.

[7] Quesada-Puga C, Izquierdo-Espin FJ, Membrive-Jiménez MJ (2024). Job satisfaction and burnout syndrome among intensive-care unit nurses: A systematic review and meta-analysis. Intensive Crit Care Nurs.

[8] Johnson JV, Hall EM (1988). Job strain, work place social support, and cardiovascular disease: A cross-sectional study of a random sample of the Swedish working population. Am J Public Health.

[9] Theorell T, Perski A, Akerstedt T (1988). Changes in job strain in relation to changes in physiological status. A longitudinal study. Scand J Work Environ Health.

[10] Siegrist J (2001). A theory of occupational stress. Stress in the Workplace: Past, Present and Future.

[11] Demerouti E, Bakker AB, Nachreiner F (2001). The job demands-resources model of burnout. J Appl Psychol.

[12] Bakker AB, Demerouti E (2017). Job demands-resources theory: Taking stock and looking forward. J Occup Health Psychol.

[13] Liu MY, Li N, Li WA (2017). Association between psychosocial stress and hypertension: A systematic review and meta-analysis. Neurol Res.

[14] Eddy P, Wertheim EH, Kingsley M (2017). Associations between the effort-reward imbalance model of workplace stress and indices of cardiovascular health: A systematic review and meta-analysis. Neurosci Biobehav Rev.

[15] Dutheil F, Baker JS, Mermillod M (2020). Shift work, and particularly permanent night shifts, promote dyslipidaemia: A systematic review and meta-analysis. Atherosclerosis.

[16] Kivimäki M, Virtanen M, Elovainio M (2006). Work stress in the etiology of coronary heart disease--A meta-analysis. Scand J Work Environ Health.

[17] Li J, Zhang M, Loerbroks A (2015). Work stress and the risk of recurrent coronary heart disease events: A systematic review and meta-analysis. Int J Occup Med Environ Health.

[18] Dragano N, Siegrist J, Nyberg ST (2017). Effort-reward imbalance at work and incident coronary heart disease: A multicohort study of 90,164 individuals. Epidemiology.

[19] Taouk Y, Spittal MJ, LaMontagne AD (2020). Psychosocial work stressors and risk of all-cause and coronary heart disease mortality: A systematic review and meta-analysis. Scand J Work Environ Health.

[20] Booth J, Connelly L, Lawrence M (2015). Evidence of perceived psychosocial stress as a risk factor for stroke in adults: A meta-analysis. BMC Neurol.

[21] Nyberg ST, Fransson EI, Heikkilä K (2014). Job strain as a risk factor for type 2 diabetes: A pooled analysis of 124,808 men and women. Diabetes Care.

[22] Sui H, Sun N, Zhan L (2016). Association between work-related stress and risk for type 2 diabetes: A systematic review and meta-analysis of prospective cohort studies. PLoS One.

[23] Li W, Yi G, Chen Z (2021). Is job strain associated with a higher risk of type 2 diabetes mellitus? A systematic review and meta-analysis of prospective cohort studies. Scand J Work Environ Health.

[24] Pena-Gralle APB, Talbot D, Duchaine CS (2022). Job strain and effort-reward imbalance as risk factors for type 2 diabetes mellitus: A systematic review and meta-analysis of prospective studies. Scand J Work Environ Health.

[25] Magnavita N, Fileni A (2014). Work stress and metabolic syndrome in radiologists: First evidence. Radiol Med.

[26] Magnavita N (2015). Work-related psychological injury is associated with metabolic syndrome components in apparently healthy workers. PLoS One.

[27] Garbarino S, Magnavita N (2015). Work stress and metabolic syndrome in police officers. A prospective study. PLoS One.

[28] Watanabe K, Sakuraya A, Kawakami N (2018). Work-related psychosocial factors and metabolic syndrome onset among workers: A systematic review and meta-analysis. Obes Rev.

[29] Kuo WC, Bratzke LC, Oakley LD (2019). The association between psychological stress and metabolic syndrome: A systematic review and meta-analysis. Obes Rev.

[30] Huang LY, Hu HY, Wang ZT (2020). Association of occupational factors and dementia or cognitive impairment: A systematic review and meta-analysis. J Alzheimers Dis.

[31] Yang T, Qiao Y, Xiang S (2019). Work stress and the risk of cancer: A meta-analysis of observational studies. Int J Cancer.

[32] Milner A, Witt K, LaMontagne AD (2018). Psychosocial job stressors and suicidality: A meta-analysis and systematic review. Occup Environ Med.

[33] van der Molen HF, Nieuwenhuijsen K, Frings-Dresen MHW (2020). Work-related psychosocial risk factors for stress-related mental disorders: An updated systematic review and meta-analysis. BMJ Open.

[34] Yang B, Wang Y, Cui F (2018). Association between insomnia and job stress: A meta-analysis. Sleep Breath.

[35] Garbarino S, Magnavita N (2019). Sleep problems are a strong predictor of stress-related metabolic changes in police officers. A prospective study. PLoS One.

[36] Marcatto F, Ferrante D, Paliga M (2025). Behavioral dysregulation at work: A moderated mediation analysis of sleep impairment, work-related stress, and substance use. AIMS Public Health.

[37] Theorell T, Hammarström A, Aronsson G (2015). A systematic review including meta-analysis of work environment and depressive symptoms. BMC Public Health.

[38] Rugulies R, Aust B, Madsen IE (2017). Effort-reward imbalance at work and risk of depressive disorders. A systematic review and meta-analysis of prospective cohort studies. Scand J Work Environ Health.

[39] Milner A, Scovelle AJ, King TL (2019). Exposure to work stress and use of psychotropic medications: A systematic review and meta-analysis. J Epidemiol Community Health.

[40] Thielmann B, Hartung J, Böckelmann I (2022). Objective assessment of mental stress in individuals with different levels of effort reward imbalance or overcommitment using heart rate variability: A systematic review. Syst Rev.

[41] Planert J, Klucken T, Finke JB (2023). Associations between hair cortisol and subjective stress measures in a large occupational sample. Psychoneuroendocrinology.

[42] Eddy P, Heckenberg R, Wertheim EH (2016). A systematic review and meta-analysis of the effort-reward imbalance model of workplace stress with indicators of immune function. J Psychosom Res.

[43] Eddy P, Wertheim EH, Hale MW (2023). A systematic review and revised meta-analysis of the effort-reward imbalance model of workplace stress and hypothalamic-pituitary-adrenal axis measures of stress. Psychosom Med.

[44] Lodahl TM, Kejnar M (1965). The definition and measurement of job involvement. J Appl Psychol.

[45] Porter LW, Lawler EE (1965). Properties of organization structure in relation to job attitudes and job behavior. Psychol Bull.

[46] Shimazu A, Schaufeli WB, Kamiyama K (2015). Workaholism vs. work engagement: The two different predictors of future well-being and performance. Int J Behav Med.

[47] Ehsan A, Klaas HS, Bastianen A (2019). Social capital and health: A systematic review of systematic reviews. SSM Popul Health.

[48] Nolzen N (2018). The concept of psychological capital: A comprehensive review. Manag Rev Q.

[49] Magnavita N, Chiorri C (2022). Development and validation of a new measure of work annoyance using a psychometric network approach. Int J Environ Res Public Health.

[50] Kawada M, Shimazu A, Miyanaka D (2024). Boredom and engagement at work: Do they have different antecedents and consequences?. Ind Health.

[51] Siegrist J, Starke D, Chandola T (2004). The measurement of effort-reward imbalance at work: European comparisons. Soc Sci Med.

[52] Siegrist J (2000). Place, social exchange and health: Proposed sociological framework. Soc Sci Med.

[53] Braunheim L, Dragano N, Khachatryan K (2024). The effects of effort-reward imbalance on the job, overcommitment, and income on life satisfaction in Germany from a longitudinal perspective. Soc Sci Med.

[54] Bakker AB, Killmer CH, Siegrist J (2000). Effort–reward imbalance and burnout among nurses. J Adv Nurs.

[55] Calnan M, Wainwright D, Almond S (2000). Job strain, effort-reward imbalance and mental distress: A study of occupations in general medical practice. Work Stress.

[56] Chen SW, Pikhart H, Peasey A (2022). Work stress, overcommitment personality and alcohol consumption based on the Effort-Reward Imbalance model: A population-based cohort study. SSM Popul Health.

[57] Porru F, Robroek SJW, Bültmann U (2021). Mental health among university students: The associations of effort-reward imbalance and overcommitment with psychological distress. J Affect Disord.

[58] Siegrist J, Li J (2016). Associations of extrinsic and intrinsic components of work stress with health: A systematic review of evidence on the effort-reward imbalance model. Int J Environ Res Public Health.

[59] Kusik D, Tokarz A, Kłosowska J (2024). Antecedents of workaholism and work engagement: A motivational perspective in research on heavy work involvement. Psychol Rep.

[60] Kahn WA (1990). Psychological conditions of personal engagement and disengagement at work. Acad Manag J.

[61] Huang SYB, Huang CH, Chang TW (2022). A new concept of work engagement theory in cognitive engagement, emotional engagement, and physical engagement. Front Psychol.

[62] Duh-Leong C, Dreyer BP, Huang TT (2021). Social capital as a positive social determinant of health: A narrative review. Acad Pediatr.

[63] Coppe T, Thomas L, Pantić N (2022). The use of social capital in teacher research: A necessary clarification. Front Psychol.

[64] Janssens H, Braeckman L, Vlerick P (2018). The relation between social capital and burnout: A longitudinal study. Int Arch Occup Environ Health.

[65] Bamford J, Klabbers G, Curran E (2021). Social capital and mental health among black and minority ethnic groups in the UK. J Immigr Minor Health.

[66] Paliga M, Kożusznik B, Pollak A (2022). The relationships of psychological capital and influence regulation with job satisfaction and job performance. PLoS One.

[67] Jayaraman A, Ramu P, Rajan SC (2023). Data driven analysis of social capital in Farmer Producer Companies. Heliyon.

[68] Firouzbakht M, Tirgar A, Oksanen T (2018). Workplace social capital and mental health: A cross-sectional study among Iranian workers. BMC Public Health.

[69] Pattussi MP, Olinto MT, Canuto R (2016). Workplace social capital, mental health and health behaviors among Brazilian female workers. Soc Psychiatry Psychiatr Epidemiol.

[70] Magnavita N, Chiorri C, Chirico F (2025). Individual work attitudes and work ability. Eur J Investig Health Psychol Educ.

[71] Kouvonen A, Kivimäki M, Vahtera J (2006). Psychometric evaluation of a short measure of social capital at work. BMC Public Health.

[72] Siegrist J, Starke D, Chandola T (2004). The measurement of effort-reward imbalance at work: European comparisons. Soc Sci Med.

[73] Magnavita N (2007). Two tools for health surveillance of job stress: The Karasek job content questionnaire and the Siegrist effort reward imbalance questionnaire. G Ital Med Lav Ergon.

[74] Siegrist J, Wege N, Puhlhofer F (2009). A short generic measure of work stress in the era of globalization: Effort-reward imbalance. Int Arch Occup Environ Health.

[75] Balducci C, Fraccaroli F, Schaufeli WB (2010). Psychometric properties of the italian version of the Utrecht Work Engagement Scale (UWES-9). Eur J Psychol Assess.

[76] Schaufeli WB, Bakker AB, Salanova M (2006). The Measurement of work engagement with a short questionnaire: A cross-national study. Educ Psychol Meas.

[77] Magnavita N, Garbarino S, Siegrist J (2012). The use of parsimonious questionnaires in occupational health surveillance. Psychometric properties of the short Italian version of the effort/reward imbalance questionnaire. Sci World J.

[78] Lumley T, Diehr P, Emerson S (2002). The importance of the normality assumption in large public health data sets. Annu Rev Public Health.

[79] Rusli BN, Edimansyah BA, Naing L (2008). Working conditions, self-perceived stress, anxiety, depression and quality of life: A structural equation modelling approach. BMC Public Health.

[80] Lewandowski CA (2003). Organizational factors contributing to worker frustration: The precursor to burnout. J Soc Soc Welfare.

[81] Yu F, Raphael D, Mackay L (2019). Personal and work-related factors associated with nurse resilience: A systematic review. Int J Nurs Stud.

[82] Thielmann B, Schwarze R, Böckelmann I (2023). A systematic review of associations and predictors for job satisfaction and work engagement in prehospital emergency medical services-challenges for the future. Int J Environ Res Public Health.

[83] Adanaqué-Bravo I, Escobar-Segovia K, Gómez-Salgado J (2023). Relationship between psychological distress, burnout and work engagement in workers during the COVID-19 pandemic: A systematic review. Int J Public Health.

[84] Cohen C, Pignata S, Bezak E (2023). Workplace interventions to improve well-being and reduce burnout for nurses, physicians and allied healthcare professionals: A systematic review. BMJ Open.

[85] Aydogdu ALF (2024). Work engagement among nurses in the context of the COVID-19 pandemic: A systematic review. Nurs Ethics.

[86] Hetzel-Riggin MD, Swords BA, Tuang HL (2020). Work engagement and resiliency impact the relationship between nursing stress and burnout. Psychol Rep.

[87] Landells EM, Albrecht SL (2019). Perceived organizational politics, engagement, and stress: The mediating influence of meaningful work. Front Psychol.

[88] Bartlett L, Buscot MJ, Bindoff A (2021). Mindfulness is associated with lower stress and higher work engagement in a large sample of MOOC participants. Front Psychol.

[89] Heinrichs K, Schultz K, Hummel S (2022). Asthma self-management at work, asthma morbidity, and the subjective prognosis of gainful employment - the role of work engagement and overcommitment: A cross-sectional study. J Asthma.

[90] Mori K, Odagami K, Inagaki M (2024). Work engagement among older workers: A systematic review. J Occup Health.

[91] Joksimovic L, Starke D, van de Knesebeck O (2002). Perceived work stress, overcommitment, and self-reported musculoskeletal pain: A cross-sectional investigation. Int J Behav Med.

[92] Coelho D, Yamaguchi S, Harb A (2024). Effort-reward and overcommitment at work and psychiatric symptoms in healthcare professionals: The mediation role of allostatic load. Compr Psychoneuroendocrinol.

[93] Håkansson C, Gard G, Lindegård A (2020). Perceived work stress, overcommitment, balance in everyday life, individual factors, self-rated health and work ability among women and men in the public sector in Sweden - A longitudinal study. Arch Public Health.

[94] Tseng PC, Lin PY, Liang WM (2022). Effort-reward imbalance and job strain index associated with health-related quality of life for civil servants in a national survey: The mediation effect of job support and over-commitment. Int J Occup Med Environ Health.

[95] Qi X, Deng H, Zhang H (2024). Over-commitment positively predicts hair cortisol concentrations only in nurses with high need for recovery. Int Arch Occup Environ Health.

[96] Bereznowski P, Atroszko PA, Konarski R (2023). Work addiction, work engagement, job burnout, and perceived stress: A network analysis. Front Psychol.

[97] Hinsch DM, Spanier K, Radoschewski FM (2018). Associations between overcommitment, effort-reward imbalance and mental health: Findings from a longitudinal study. Int Arch Occup Environ Health.

[98] Gächter M, Savage DA, Torgler B (2011). The relationship between stress, strain and social capital. Policing.

[99] Ommen O, Driller E, Köhler T (2009). The relationship between social capital in hospitals and physician job satisfaction. BMC Health Serv Res.

[100] Jutengren G, Jaldestad E, Dellve L (2020). The potential importance of social capital and job crafting for work engagement and job satisfaction among health-care employees. Int J Environ Res Public Health.

[101] Bakker AB, Emmerik HV, Euwema MC (2006). Crossover of burnout and engagement in work teams. Work Occupation.

[102] Kouvonen A, Oksanen T, Vahtera J (2008). Low workplace social capital as a predictor of depression: The Finnish public sector study. Am J Epidemiol.

[103] Villalonga-Olives E, Almansa J, Shaya F (2020). Perceived social capital and binge drinking in older adults: The health and retirement study, US data from 2006–2014. Drug Alcohol Depend.

[104] Oliveira-Dias C, Morais S, Costa AR (2022). Sex differences in the association between social capital and healthcare use-Results from the Survey of Health, Ageing and Retirement in Europe (SHARE). Health Soc Care Community.

[105] Chang H, Zhou J, Wang Z (2023). The impact of social capital on successful ageing of empty nesters: A cross-sectional study. J Adv Nurs.

[106] Nitzsche A, Kuntz L, Miedaner F (2017). Staff working in hospital units with greater social capital experience less work-home conflict: Secondary analysis of a cross-sectional study. Int J Nurs Stud.

[107] Tsounis A, Xanthopoulou D, Kafetsios K (2024). Workplace and individual social capital as moderators on the work-life interface processes: Testing a multilevel model. Group Organ Manage.

[108] Raja U, Bouckenooghe D, Syed F (2018). Interplay between PO fit, transformational leadership and organizational social capital. Pers Rev.

[109] Tafvelin S, Nielsen K, von Thiele Schwarz U (2019). Leading well is a matter of resources: Leader vigour and peer support augments the relationship between transformational leadership and burnout. Work & Stress.

[110] Lyons JB, Schneider TR (2009). The effects of leadership style on stress outcomes. Leadership Quart.

[111] Hammond M, Cleveland JN, O'Neill JW (2015). Mediators of transformational leadership and the work-family relationship. J Manage Psychol.

[112] de Neve JE (2018). Work and well-being: A global perspective. Global happiness policy report 2018.

[113] Magnavita N (2018). Obstacles and future prospects: Considerations on health promotion activities for older workers in Europe. Int J Environ Res Public Health.

[114] Mittelmark MB, Bauer GF, Vaandrager L (2022). The Handbook of Salutogenesis.

[115] Lowensteyn I, Berberian V, Berger C (2019). The sustainability of a workplace wellness program that incorporates gamification principles: Participant engagement and health benefits after 2 years. Am J Health Promot.

[116] Gray P, Senabe S, Naicker N (2019). Workplace-based organizational interventions promoting mental health and happiness among healthcare workers: A realist review. Int J Environ Res Public Health.

[117] Ge J, He J, Liu Y (2021). Effects of effort-reward imbalance, job satisfaction, and work engagement on self-rated health among healthcare workers. BMC Public Health.

[118] Amano H, Fukuda Y, Baden MY (2020). Is work engagement associated with healthier dietary patterns? A cross-sectional study. J Occup Health.

[119] Amatori S, Gobbi E, Sisti D (2024). Physical activity, musculoskeletal disorders, burnout, and work engagement: A cross-sectional study on Italian white-collar employees. Front Public Health.

[120] Tims M, Bakker AB (2010). Job crafting: Towards a new model of individual job redesign. SA J Ind Psychol.

[121] Kossek EE, Hammer LB, Kelly EL (2014). Designing work, family, and health organizational change initiatives. Organ Dynamics.

[122] Eby LT, Robertson MM (2020). The psychology of workplace mentoring relationships. Ann Rev Organ Psychol Organ Behavior.

[123] Kizuki M, Fujiwara T (2020). Quality of supervisor behaviour, workplace social capital and psychological well-being. Occup Med (Lond).

[124] Limm H, Gündel H, Heinmüller M (2011). Stress management interventions in the workplace improve stress reactivity: A randomised controlled trial. Occup Environ Med.

[125] Shimazu A, Umanodan R, Schaufeli WB (2006). Effects of a brief worksite stress management program on coping skills, psychological distress and physical complaints: A controlled trial. Int Arch Occup Environ Health.

[126] Biron C, Karanika-Murray M (2014). Process evaluation for stress and well-being interventions: Why and how?. Int J Stress Man.

[127] Bakker AB, Demerouti E (2007). The job demands-resources model: State of the art. J Man Psychol.

[128] Magnavita N (2025). Evolution of risk acceptability. G It Psicol Med Lav.

[129] Williams JC, Berdahl JL, Vandello JA (2016). Beyond work-life “Integration”. Annu Rev Psychol.

[130] Wei JL, Villwock JA (2021). Balance versus integration: Work-life considerations. Otolaryngol Clin North Am.

